# Cell-adhesive RGD peptide-displaying M13 bacteriophage/PLGA nanofiber matrices for growth of fibroblasts

**DOI:** 10.1186/2055-7124-18-14

**Published:** 2014-10-03

**Authors:** Yong Cheol Shin, Jong Ho Lee, Linhua Jin, Min Jeong Kim, Jin-Woo Oh, Tai Wan Kim, Dong-Wook Han

**Affiliations:** Department of Cogno-Mechatronics Engineering, Pusan National University, Busan, 609-735 Korea; Department of Nanomaterials Engineering, College of Nanoscience & Nanotechnology, Pusan National University, Busan, 609-735 Korea; Department of Design, College of Arts, Pusan National University, Busan, 609-735 Korea

**Keywords:** M13 bacteriophages, RGD peptide, PLGA nanofibers, Extracellular matrices, Fibroblasts

## Abstract

**Background:**

M13 bacteriophages can be readily fabricated as nanofibers due to non-toxic bacterial virus with a nanofiber-like shape. In the present study, we prepared hybrid nanofiber matrices composed of poly(lactic-*co*-glycolic acid, PLGA) and M13 bacteriophages which were genetically modified to display the RGD peptide on their surface (RGD-M13 phage).

**Results:**

The surface morphology and chemical composition of hybrid nanofiber matrices were characterized by scanning electron microscopy (SEM) and Raman spectroscopy, respectively. Immunofluorescence staining was conducted to investigate the existence of M13 bacteriophages in RGD-M13 phage/PLGA hybrid nanofibers. In addition, the attachment and proliferation of three different types of fibroblasts on RGD-M13 phage/PLGA nanofiber matrices were evaluated to explore how fibroblasts interact with these matrices. SEM images showed that RGD-M13 phage/PLGA hybrid matrices had the non-woven porous structure, quite similar to that of natural extracellular matrices, having an average fiber diameter of about 190 nm. Immunofluorescence images and Raman spectra revealed that RGD-M13 phages were homogeneously distributed in entire matrices. Moreover, the attachment and proliferation of fibroblasts cultured on RGD-M13 phage/PLGA matrices were significantly enhanced due to enriched RGD moieties on hybrid matrices.

**Conclusions:**

These results suggest that RGD-M13 phage/PLGA matrices can be efficiently used as biomimetic scaffolds for tissue engineering applications.

## Background

In the body, most cells grow on the extracellular matrix (ECM) which is porous structure consisting of fibrous proteins and glycosaminoglycans. The ECM is the outer parts of cells in tissues and provides an essential environment for cells to survive. The ECM is very important because it affects cellular behaviors such as cell adhesion, proliferation and differentiation through biochemical or physical signal as well as provides a 3D microenvironment conductive to cellular growth [1–4]. Recently, many efforts are under way to develop a biomimetic artificial ECM for tissue engineering applications by using various biodegradable polymers [5–7]. Poly(lactic-co-glycolic acid, PLGA), as one of synthetic biodegradable polymers, has been most extensively used in drug delivery and scaffold applications due to its excellent biocompatibility and suitable physicochemical properties [8–11]. Among many biomolecules within the ECM, adhesive proteins such as fibronectin and laminin play an important role in cellular attachment and proliferation. Generally, most of animal cells attach to the ECM through direct interaction between transmembrane integrin receptor and adhesive proteins. The primary sequence motif of fibronectin for integrin binding is a tripeptide, Arg-Gly-Asp (RGD [12–14]. Therefore, matrices which are enriched with RGD peptides promote not only cellular attachment but also cellular behaviors [15, 16].

An electrospinning technique has been widely used to fabricate nanofibrous matrices which were structurally similar to the natural ECM. A previous study has already shown that nanofibrous matrices fabricated by an electrospinning process were suitable for tissue engineering scaffolds [17]. The advantage of this technique is that non-woven porous matrices can be easily produced as well as incorporated with bioactive agents including growth factors, peptides and drugs [7].

M13 bacteriophage is bacterial virus with nanofiber-like shaped [18, 19]. Moreover, M13 bacteriophages do not have any detrimental effect on mammalian cells [20–22]. Some previous studies showed that M13 bacteriophage could express many desired proteins on their surface [23–25]. In addition, M13 bacteriophages can be readily fabricated as nanofibers via an electrospinning process due to their structural property [26].

The aim of the present study was to fabricate RGD peptide-displaying M13 bacteriophages (RGD-M13 phages)/PLGA hybrid nanofiber matrices to mimic the natural ECM by electrospinning. We showed that RGD-M13 phage/PLGA hybrid matrices were successfully fabricated and RGD-M13 phages were uniformly distributed over the matrices. Furthermore, we examined cellular behaviors of fibroblasts on RGD-M13 phage/PLGA nanofiber matrices to explore how the cells interact with these matrices. As a result, we demonstrated that these matrices improved cellular behaviors by synergistic effects, resulting from the excellent biocompatibility of PLGA and the ability to enhance the cell attachment of RGD sequences displayed on the surface of M13 bacteriophages. Therefore, this result implies that RGD-M13 phage/PLGA hybrid matrices can be one of the best potential candidates for biomimetic tissue engineering scaffolds.

## Methods

### Fabrication of RGD-M13 phage/PLGA nanofibers by electrospinning

**Figure 1:**
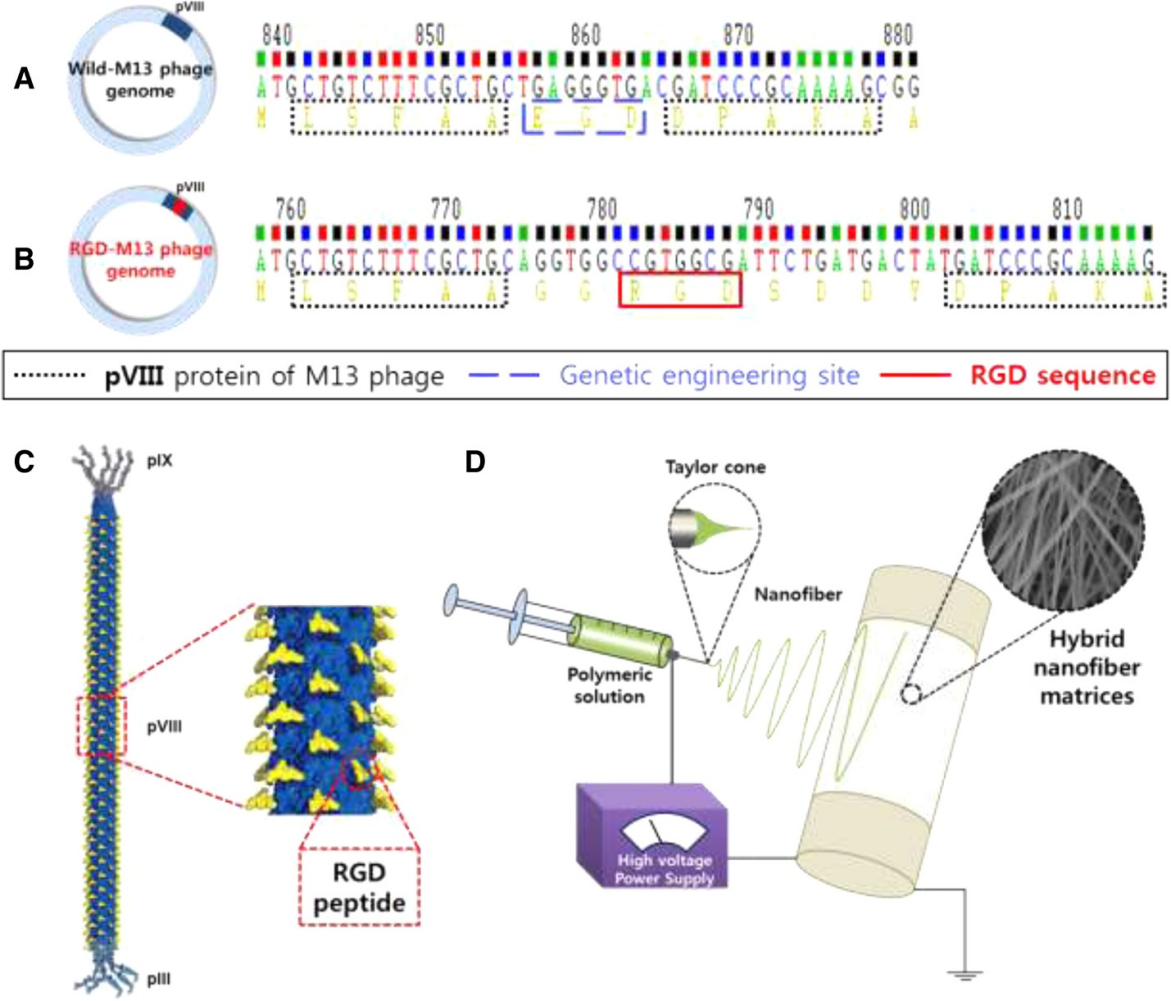
**Schematic diagrams of RGD peptide-displaying M13 bacteriophage and electrospinning process for fabrication of hybrid nanofibers. (A)** DNA sequence of wild-type M13 bacteriophages. **(B)** DNA sequence of RGD peptide-displaying M13 bacteriophages (RGD-M13 phages). **(C)** Structure of genetically engineered RGD-M13 phage. **(D)** Fabrication of RGD-M13 phage/PLGA nanofibers by electrospinning.

Genetic engineering of M13 bacteriophage was conducted according to the method as previously described [20]. In brief, to display a desired peptide on major coat protein of M13 bacteriophage surface, an inverse PCR cloning method was carried out as described elsewhere [24, 27, 28]. As shown in Figure 1A-C, we built RGD-M13 phages through genetic modification of wild-type M13 bacteriophages. PLGA resins (PLA/PGA = 75/25, MW = 70–110 kDa) were purchased from Sigma-Aldrich (St Louis, MO). RGD-M13 phage/PLGA nanofiber matrices were fabricated by an electrospinning process (Figure 1D) as described elsewhere [29]. Random oriented RGD-M13 phage/PLGA nanofibers were collected on a steel rotating wheel covered with an aluminum foil. Then, electrospun nanofiber matrices were dried overnight under vacuum at room temperature in order to remove any residual solvent.

### Characterizations of RGD-M13 phage/PLGA hybrid nanofibers

The surface morphology of RGD-M13 phage/PLGA matrices was observed under a scanning electron microscope (SEM). The matrices were coated with an ultrathin layer of gold/platinum by an ion sputter (E1010, Hitach, Tokyo, Japan) prior to analysis. The RGD-M13 phages and nanofibers were characterized by using Raman spectroscopy. For immunofluorescence staining to examine whether M13 bacteriophages exist in RGD-M13 phage/PLGA matrices or not, the matrices were incubated with primary anti-M13 bacteriophage antibody [at 1:250 in 2% bovine serum albumin (BSA) solution in phosphate-buffered saline (PBS, pH 7.4); Sigma-Aldrich] for 2 h at room temperature and then incubated with secondary FITC-conjugated goat anti-rabbit IgG (at 1:500 in 2% BSA solution in PBS; Abcam Inc., Cambridge, MA) for 1 h at room temperature. Immunostained matrices were imaged with an Olympus IX81 inverted fluorescence microscope (Olympus Optical Co., Osaka, Japan).

### Cells and culture conditions

In the present study, three different types of fibroblasts were employed – two types were immortalized and cancerous cell lines and one was a primary cell. Murine fibroblasts (L-929 cells) and human fibrosarcoma cells (HT-1080 cells) were purchased from American Type Culture Collection (ATCC, Rockville, MD) and routinely maintained in Dulbecco’s modified Eagle’s Medium (DMEM, Welgene, Daegu, Korea). Human dermal fibroblasts (HDFs) from neonatal dermis were kindly provided by Dr. Dong Kyun Rah (Department of Plastic and Reconstructive Surgery, Yonsei University College of Medicine, Seoul, Korea) and cultured in fibroblast basal medium (FBM, Lonza, Basel, Switzerland). All cells were cultured in complete media supplemented with 10% fetal bovine serum (FBS, Welgene) and 1% antibiotic-antimycotic solution (including 10,000 units penicillin, 10 mg streptomycin and 25 μg amphotericin B per ml, Sigma-Aldrich, St. Louis, MO) at 37°C in a humidified atmosphere containing 5% CO_2_.

### *In vitro*assays for fibroblast behaviors on RGD-M13 phage/PLGA nanofibers

Cell attachment and proliferation were measured by using a cell counting kit-8 (CCK-8, Dojindo, Kumamoto, Japan), which contains highly water-soluble tetrazolium salt [WST-8, 2-(2-methoxy-4-nitrophenyl)-3-(4-nitrophenyl)-5-(2,4-disulfophenyl)-2*H*-tetrazolium, monosodium salt], reduced to a water-soluble formazan dye by dehydrogenases in cells. The number of viable cells was found to be directly proportional to the metabolic reaction products obtained in the CCK-8 assay [30]. Typically, three types of fibroblasts were seeded at a density of 1 × 10^4^ cells/mL on PLGA or RGD-M13 phage/PLGA matrices. According to the manufacturer’s instruction, each cell culture was incubated with a WST-8 solution in the last 4 hours of culture periods for cell attachment (6 hours) or proliferation (1, 3, 5 and 7 days) at 37°C in the dark. Parallel sets of cells cultured on tissue culture plastics (TCP) were regarded as controls. The absorbance was determined at 450 nm by an ELISA reader (SpectraMax 340, Molecular Device Co., Sunnyvale, CA). For morphological observations, HDFs cultured on matrices for 3 days were fixed with 3.7% formaldehyde solution (Sigma-Aldrich) for 10 minutes. After fixation, HDF-cultured matrices were dehydrated in a graded series of ethanol, followed by air-drying overnight. The matrices were sputter-coated with gold/platinum and then observed under a scanning electron microscope (SEM, Hitachi S-800, Tokyo, Japan) at an accelerating voltage of 5 kV.

### Statistical analysis

All variables were tested in three independent cultures for each experiment, which was repeated twice (n = 6). Quantitative data are expressed as the mean ± standard deviation (SD). Data were tested for homogeneity of variances using the test of Levene, prior to statistical analysis. Multiple comparisons to detect the cellular behaviors on RGD-M13 phage/PLGA nanofiber matrices were carried out using one-way analysis of variance (ANOVA, SAS Institute, Cary, NC), which was followed by the Bonferroni test when variances were homogeneous and the Tamhane test when variances were not. A value of *p* < 0.05 was considered statistically significant.

## Results and discussion

### Physicochemical characteristics of RGD-M13 phage/PLGA hybrid nanofibers

**Figure 2:**
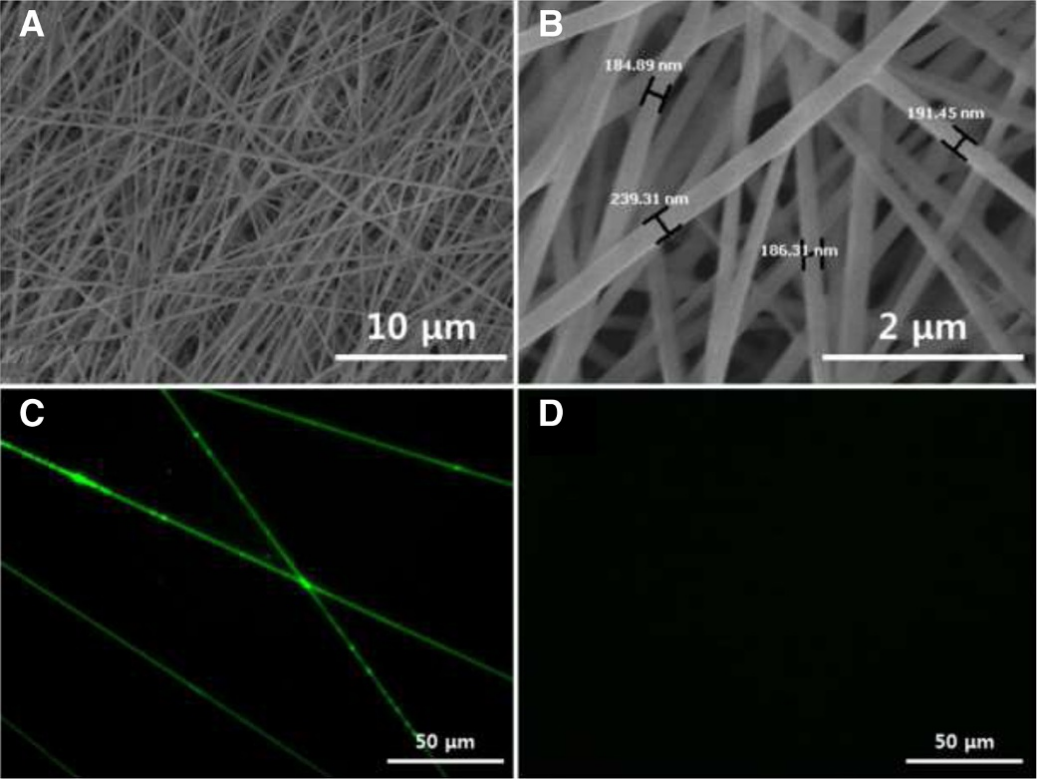
**Surface morphology and immunostaining of electrospun nanofibers. (A, B)** SEM images of RGD-M13 phage/PLGA nanofibers. Fluorescence microscopic images of RGD-M13 phage/PLGA nanofibers **(C)** and pure PLGA nanofibers **(D)**. RGD-M13 phages in RGD-M13 phage/PLGA nanofibers were immunostained with FITC-labeled anti-M13 phage antibody and fluoresced green.

As shown in Figure 1, RGD-M13 phages and PLGA were blended and then fabricated into hybrid nanofibers thru an electrospinning technique. The artificial scaffolds should be similar to structural property of the natural ECM and support cell growth through 3D microenvironment. As shown in Figure 2A, the morphology of RGD-M13 phage/PLGA nanofibers was not only non-woven porous but also uniform and bead-less as similar to the natural ECM. The RGD-M13 phage/PLGA nanofibers had an average diameter of 190 ± 30 nm (Figure 2B). The RGD-M13 phages of electrospun nanofibers could be very well interaction with cells because the matrices consisted of nanometer scale fibers had a very high surface area-to-volume ratio. Immunostaining fluorescence image showed that genetically engineered RGD-M13 phages were located on the hybrid nanofibers (Figure 2C). Green fluorescence from RGD-M13 phages labeled with FITC was detected along the RGD-M13 phage/PLGA nanofibers. On the contrary, any fluorescence was not exhibited from pure PLGA nanofibers (Figure 2D).

**Figure 3:**
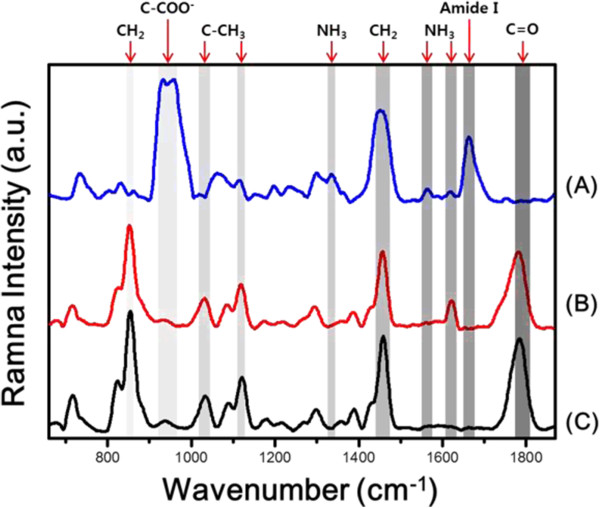
**Compositional analysis of electrospun nanofibers by Raman spectroscopy.** Raman spectra of RGD-M13 phages **(A)**, RGD-M13 phage/PLGA nanofibers **(B)** and pure PLGA nanofibers **(C)**.

Raman spectroscopy was used to evaluate the composition of the nanofibers. The Raman spectra of RGD-M13 phages, RGD-M13 phage/PLGA nanofibers and pure PLGA nanofibers were shown in Figure 3. There are many bands representing the PLGA. The strong bands observed at 850 and 1460 cm^-1^ were assigned to CH_2_ [31]. Another band observed at 1780 cm^-1^ represents the C = O stretch of the ester group [32]. The spectra of PLGA were more predominant than that of RGD-M13 phages because the ratio of PLGA was much greater than that of RGD-M13 phages in nanofibers. However, there is a specific peak at 1620 cm^-1^ which can be attributed to bending vibration of NH_3_^+^ [33]. This peak was derived from amino acids of the RGD-M13 phages. In addition, a characteristic peak was found near 930 cm^-1^ which is assigned to the C-COO^-^ stretch from carboxylate group of glycine [31]. These results imply that RGD-M13 phages were existed in the nanofibers. From immunofluorescence staining and Raman spectroscopy, it was demonstrated that RGD-M13 phages were well incorporated into electrospun nanofibers.

### Fibroblast behaviors on RGD-M13 phage/PLGA nanofiber matrices

**Figure 4:**
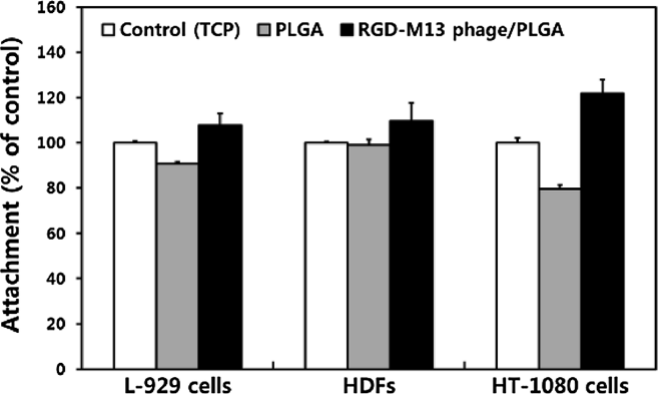
**Cell attachment of L-929 cells, HDFs and HT-1080 cells on RGD-M13 phage/PLGA nanofiber matrices.** Cell attachment was determined by a CCK-8 assay. Data are expressed as mean ± SD based on at least duplicate observations from three independent experiments.

**Figure 5:**
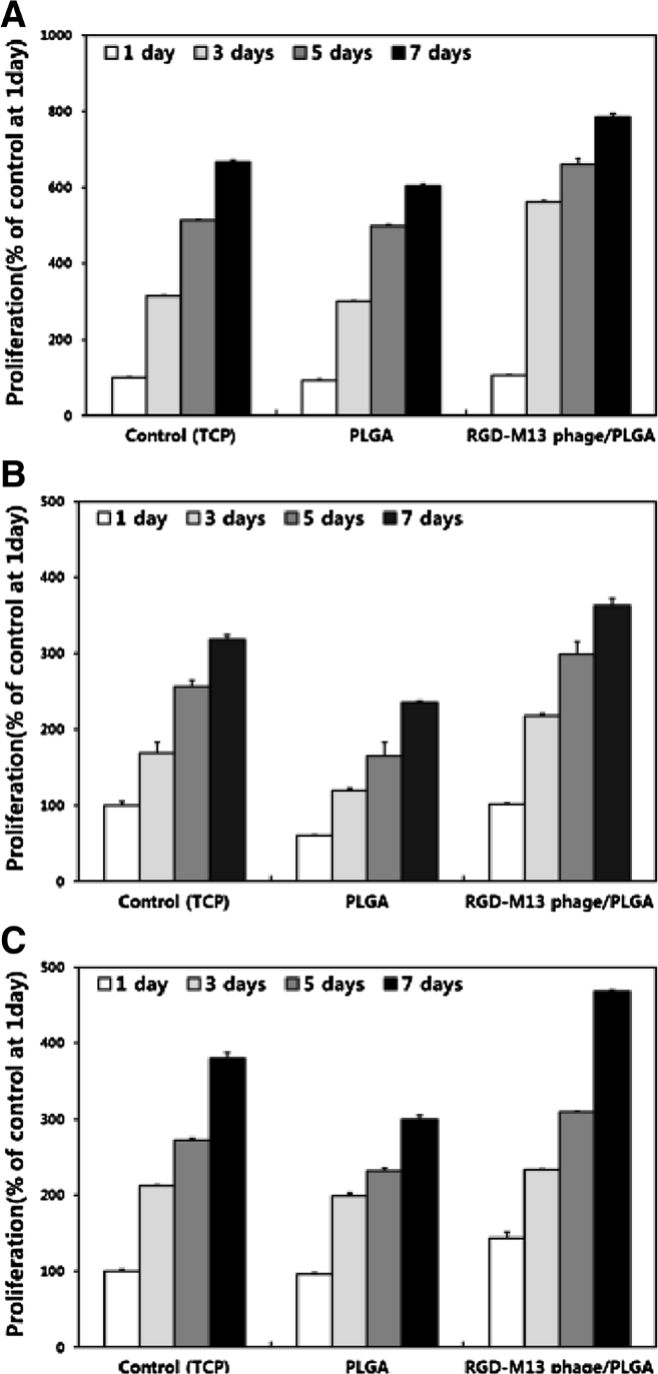
**Cell proliferation of fibroblasts cultured on RGD-M13 phage/PLGA nanofiber matrices for 1 – 7 days.** Proliferation of L-929 cells **(A)**, HDFs **(B)** and HT-1080 cells **(C)** were determined by a CCK-8 assay. Data are expressed as mean ± SD based on at least duplicate observations from three independent experiments.

We examined cellular behaviors of fibroblasts on the RGD-M13 phage/PLGA nanofiber matrices. In this study, three types of fibroblast were used: murine fibroblast cell lines (L-929 cells), primary human dermal fibroblasts (HDFs), and human fibrosarcoma cell lines (HT-1080 cells). The cell viability was measured to assess a cell attachment when cells had been seed on the matrices for 6 hours. As shown in Figure 4, fibroblasts showed the highest adhesion where they were cultured on RGD-M13 phage/PLGA matrices regardless of cell types. As mentioned above, RGD peptides contained the matrices have contributed to the enhancement of cell adhesion. In contrast, cell adhesion on pure PLGA matrices was decreased because the PLGA matrices did not contain any materials which could improve cell adhesion. In addition, we evaluated the proliferation of fibroblasts on pure PLGA and RGD-M13 phage/PLGA matrices. As shown in Figure 5, the proliferation of fibroblasts was increased in a time-dependent manner. However, fibroblasts cultured on the RGD-M13 phage/PLGA matrices showed the highest proliferation as compared with that on pure PLGA matrices and even the control. These results implied that the RGD peptides effectively promote not only initial attachment but also proliferation of fibroblasts to the matrices. In addition, proliferation of both cell lines and primary cells was increased. These results correspond well with previous studies. It has been reported that RGD-functionalized substrates are able to promote cell attachment [34, 35]. It is suggested that RGD peptides enhance cellular behaviors regardless of the cell types.

**Figure 6:**
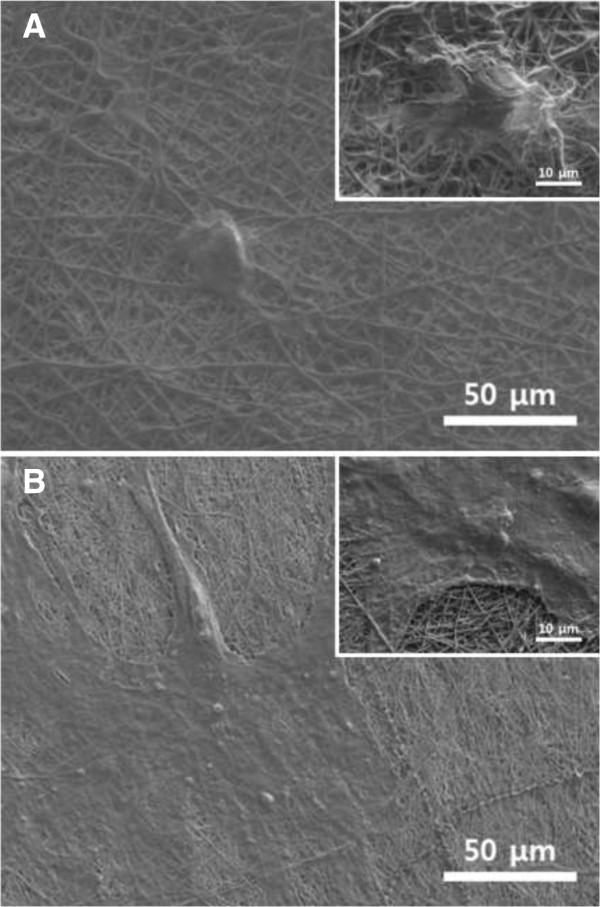
**Cell morphology of HDFs grown on electrospun nanofiber matrices.** SEM images of HDFs cultured on pure PLGA nanofiber matrices **(A)** and RGD-M13 phage/PLGA nanofiber matrices **(B)** for 3 days (magnification: in A and C, × 500 and in B and D, × 2,000). All photographs shown in this figure are representative of six independent experiments with similar results.

SEM images of HDFs cultured on the pure PLGA and RGD-M13 phage/PLGA matrices were presented on Figure 6. As shown in Figure 6A, HDFs cultured on pure PLGA matrix showed abnormal morphologies because the cells could not properly attach to the matrices. In contrast, HDFs cultured on hybrid matrices presented typical and well-spread morphology (Figure 6B). This result indicated that the cells were effectively attached and grown on the hybrid matrices. Previous reports documented that the RGD peptides containing scaffolds enhance cellular activities of various cell types including attachment, proliferation and differentiation [36–38]. Moreover, the hybrid matrices were good at support cellular growth without losing their nanofibrous structure even in the cell culture environment. Therefore, it is demonstrated that the hybrid matrices are biofunctional scaffolds which can improve cellular behaviors as well as induce cellular attachment.

## Conclusions

This study was designed to fabricate RGD-M13 phage/PLGA hybrid matrices that could applied for tissue engineering scaffolds. The suitable scaffold should be biocompatible and biodegradable. In addition, its structure should have a porous structure which is analogous to the natural ECM in order to support the growth of cells. We successfully fabricated the RGD-M13 phage/PLGA matrices by using electrospinning and analyzed various properties of them. Results showed that the hybrid matrices had outstanding biocompatibility and suitable physicochemical properties as well as structurally comparable to the natural ECM. Moreover, they were biofunctional scaffolds which could effectively improve cellular attachment and proliferation through RGD peptides existed along the nanofibers. Based on these results, RGD-M13 phage/PLGA hybrid matrices had a high probability to use for biodegradable scaffolds. In summary, it is suggested that the RGD-M13 phage/PLGA hybrid matrices can be employed to bioactive scaffolds for tissue engineering application.

## Availability of supporting data

There was no available supporting data.
